# MRI Screening for Chronic Anticoagulation in Atrial Fibrillation

**DOI:** 10.3389/fneur.2013.00137

**Published:** 2013-10-04

**Authors:** Mark Fisher

**Affiliations:** ^1^Departments of Neurology, Anatomy & Neurobiology, and Pathology & Laboratory Medicine, UC Irvine School of Medicine, Irvine, CA, USA

**Keywords:** atrial fibrillation, stroke, microbleeds, anticoagulation, MRI, hemorrhage

## Abstract

Anticoagulation is highly effective in preventing stroke due to atrial fibrillation, but numerous studies have demonstrated low utilization of anticoagulation for these patients. Assessment of clinicians’ attitudes on this topic indicate that fear of intracerebral hemorrhage (ICH), rather than appreciation of anticoagulation benefits, largely drives clinical decision-making for treatment with anticoagulation in atrial fibrillation. Risk stratification strategies have been used for anticoagulation benefits and hemorrhage risk, but ICH is not specifically addressed in the commonly used hemorrhage risk stratification systems. Cerebral microbleeds are cerebral microscopic hemorrhages demonstrable by brain MRI, indicative of prior microhemorrhages, and predictive of future risk of ICH. Prevalence of cerebral microbleeds increases with age; and cross-sectional and limited prospective studies generally indicate that microbleeds confer substantial risk of ICH in patients treated with chronic anticoagulation. MRI thus is a readily available and appealing modality that can directly assess risk of future ICH in patients receiving anticoagulants for atrial fibrillation. Incorporation of MRI into routine practice is, however, fraught with difficulties, including the uncertain relationship between number and location of microbleeds and ICH risk, as well as cost-effectiveness of MRI. A proposed algorithm is provided, and relevant advantages and disadvantages are discussed. At present, MRI screening appears most appropriate for a subset of atrial fibrillation patients, such as those with intermediate stroke risk, and may provide reassurance for clinicians whose concerns for ICH tend to outweigh benefits of anticoagulation.

## Introduction

Stroke prevention relies on chronic anticoagulation in an increasingly selective fashion. Well-constructed clinical trials have led to a nearly exclusive focus on chronic anticoagulation for prevention of cardiogenic stroke, specifically stroke prevention in patients with atrial fibrillation. Given the well-known high morbidity and mortality of cardiogenic stroke and the high degree of preventability of these events, one might assume that clinicians are highly motivated to aggressively initiate anticoagulation for patients with atrial fibrillation in an attempt to eliminate this most devastating consequence of that arrhythmia. But this assumption may not be correct; anticoagulation is underutilized in this population.

This article will review issues underlying this underutilization of anticoagulation, the reasons for avoidance of highly effective treatment for a devastating disorder, and offer a possible way out of this predicament with better targeting of patients likely to benefit from this treatment. An important issue implicit in this clinical problem is the coexistence of ischemic and hemorrhagic cerebrovascular disease, necessitating new diagnostic terminologies. This paper will explore the potential benefits of using high quality brain imaging for risk stratification for anticoagulant-associated intracerebral hemorrhage (ICH), and discuss how this may be incorporated into clinical practice in a manner that will lead to substantial improvements in stroke prevention.

## Underutilization of Anticoagulation for Atrial Fibrillation

Precise numbers regarding anticoagulation utilization can be difficult to obtain from the literature, due to the necessity for distinguishing patients receiving anticoagulants and then discontinuing usage, vs. those patients who continued to take anticoagulants for a prolonged period of time. One careful study showed increasing warfarin utilization through the survey period of 1992–2002, peaking at 56.3% in 2002, with 59.9% usage in patients lacking risk factors for falls and bleeding (1). A follow-up study reported continued rise in warfarin utilization, peaking at 63.1% in 2007 (2).

Other studies have been less encouraging. For example, a survey of anticoagulant use world-wide reported that half of the analyzed studies (15/29) described treatment with anticoagulation in less than half of high risk atrial fibrillation patients (3), while more than half of the studies (21/29) reported anticoagulation usage in <60% of patients with prior stroke or transient ischemic attack (3). A recent Canadian study reported usage of anticoagulation in 49% of older patients (aged 65 and older) with atrial fibrillation, with no relationship between anticoagulation use and stroke risk (4). Among more than 1,100 patients in an American primary care population (mean age 70 years), 67% were treated with anticoagulants, but treatment was discontinued in 25.4% within 1 year and at end of follow-up period (mean 3.4 years) 45.7% remained on warfarin (5). Inappropriate discontinuation of anticoagulation appears to be a significant problem, and is associated with excessive thrombotic risk (6). A survey of more than 13,000 American nursing home residents revealed warfarin usage in 30% of patients with atrial fibrillation (7). Another recent analysis, of more than 170,000 patients, reported that warfarin was prescribed for a similar proportion of patients regardless of whether their stroke risk was low (40.1%), medium (43.5%), or high (42.1%); moreover, only 32.8% of patients received 6 months of uninterrupted warfarin therapy for stroke prevention (8).

This rather robust literature on use of anticoagulation for atrial fibrillation patients thus shows substantial variation. Utilization of anticoagulation varies from a high end just over 60%, to a low end of 30% in nursing homes. At the very least, there is considerable room for improvement.

## Effectiveness of Anticoagulation for Prevention of Cardiogenic Stroke

The low utilization of anticoagulation for atrial fibrillation patients could perhaps be understood in the context of therapeutic ineffectiveness. But this is hardly the case. Individual trials as well as pooled analyses have demonstrated an estimated 65–68% stroke reduction by warfarin (9, 10). Moreover, atrial fibrillation-associated strokes are among the most devastating: compared to non-atrial fibrillation-associated strokes, there is greater severity, more disability, nearly twice likelihood to have in-hospital medical complications (43.3 vs. 24.4%), longer hospital stay (15 vs. 9 days), and more than double both short-term (30 days) mortality (14.7 vs. 5.8%) and long-term (1 year) mortality (31.7 vs. 13.7%) (11). Stroke due to atrial fibrillation is therefore among the most severe while simultaneously being among the most preventable cerebral ischemic events.

## Reasons for Underutilization: Ignorance or Fear?

Rather than bemoan the underutilization of anticoagulants for atrial fibrillation-associated stroke, a more useful approach is to attempt to understand the underlying causes of this unusual behavior among clinicians. It is typically assumed that low utilization of effective therapy reflects lack of knowledge or frank ignorance of the benefits provided by anticoagulation in this setting. However, a critical assessment of physician’s attitudes shed some light on this topic. A survey of 30 studies indicated that physicians are reluctant to prescribe anticoagulation for older patients with atrial fibrillation because of excessive concern for prior hemorrhage and fall risk (12). In a particularly well-detailed and systematic survey of clinicians’ attitudes toward anticoagulation for atrial fibrillation, there was no relationship between use of warfarin and its perceived benefits (13). On the contrary, decision-making was largely driven by fear, with an overarching concern regarding ICH as a consequence of warfarin use (13). Physicians anticipating regret over ICH from warfarin were approximately 80% less likely to recommend warfarin in case vignettes. Moreover, risks of ICH were consistently overestimated, at a rate far higher than reported in the literature (13). Thus, a principal driving force for underutilization of anticoagulation relates to concern for hemorrhage, specifically ICH, as an adverse effect. The actual risk of ICH with warfarin use in this population is typically viewed as ranging from 0.3 to 1% annual risk (14), although first year risks as high as 2.5% have been reported (15). Newer anticoagulants include dabigatran (direct thrombin inhibitor) and rivaroxaban (factor Xa inhibitor), which reportedly reduce risk of intracerebral hemorrhage by as much as one-third (rivaroxaban vs. warfarin) and 60% (dabigatran vs. warfarin) (16, 17). While it is not known how use of these newer agents impacts decision-making for anticoagulation in atrial fibrillation, it has been estimated that threshold stroke risk for triggering anticoagulation may be lowered (18). Nevertheless, reversibility of these agents has remained a concern (19).

## Risk Stratification Strategies: Success and Failure

Risk stratification paradigms are an attractive option for addressing and potentially correcting the underutilization of anticoagulation for stroke prevention in atrial fibrillation. In fact, there are now a number of these point systems available. These strategies all have their acronyms, and represent a serious attempt to provide a quick assessment of risks.

Stroke risk with atrial fibrillation has been most frequently addressed with the CHADS2 system. The simplicity of CHADS2 is appealing, with one point given for congestive heart failure, hypertension, age >75 years, diabetes, and two points for stroke or TIA. A modification of CHADS2 is the CHA2DS2-VASc system, in which stroke risk is stratified by giving points for congestive heart failure or left ventricular dysfunction, hypertension, age 75 or older (two points), diabetes, stroke (two points), vascular disease, age 65–74, and sex category (one point for females) (20). The initial CHADS2 system has the advantage of the two by virtue of its simplicity, but neither address hemorrhage risk. And as described above, decision-making for anticoagulation is substantially impacted by fear of ICH.

As an alternate or supplement to stroke risk-driven stratification systems, at least three major classifications have been used to address hemorrhage risk. The HEMORR2HAGES system gives two points for a prior hemorrhage, with one point for risk factors hepatic or renal disease, ethanol abuse, malignancy, age >75 years, reduced platelet count or function, hypertension (uncontrolled), anemia, genetic factors, excessive fall risk, and stroke (21). Aside from the relative complexity of this system, hemorrhage prediction with this system focuses largely on gastrointestinal hemorrhages (21).

A later system developed for assessing hemorrhage risk is the ATRIA paradigm, in which variable number of points are given for anemia (three points), severe renal disease (three points), age 75 or older (two points), prior bleeding (one point), and hypertension (one point) (22). This system appears to be effective for predicting overall hemorrhage risk, with rates of 0.4% for 0 points and 17.3% annual hemorrhage risk for 10 points (22). However, this system makes no attempt to differentiate ICH risk from risks of other forms of hemorrhage.

A third system, HAS-BLED has been shown to be more efficacious in hemorrhage prediction, compared to HEMORR2HAGES and ATRIA (23). HAS-BLED allocates one point each for hypertension, abnormal renal or liver function, stroke, bleeding, labile INR, elderly (>65 years), and drugs or alcohol. HAS-BLED was significantly better for hemorrhage prediction than the other two systems, but overall predictive performance for both major bleeding and clinically relevant bleeding was considered modest (23). On the other hand, HAS-BLED was the only one of the three systems that had predictive value for ICH. This latter predictive performance was considered “good,” although predictive power was only mid-way between chance and perfect discrimination for future ICH, and overall effectiveness of HAS-BLED was largely focused on bleeding events that were not considered “major” (i.e., non-ICH) (23).

In summary, these risk stratification systems have some effectiveness in both predicting atrial fibrillation-related stroke and warfarin-related hemorrhage. However, underutilization of anticoagulation appears to be less related to extent of appreciation of its stroke-preventing benefits and more driven by decision-making that has become distorted by over-emphasis on hemorrhagic risks. Moreover, the risk of the adverse event of greatest concern (ICH) is not directly addressed by the standard hemorrhage stratification systems, which primarily predict gastrointestinal hemorrhage. It would appear that a rather different approach is indicated.

## Cerebral Microbleeds as Risk-Indicators for Intracerebral Hemorrhage

Cerebral microbleeds are MRI-demonstrable signatures of small areas of brain hemorrhage. These hemorrhagic areas are seen on gradient echo and susceptibility-weighted imaging, and show a striking age-dependent increasing prevalence varying from 6.5% in individuals aged 45–50, 11.5% for ages 50–59 years, 16.8% for ages 60–69 years, and 35.7% for persons age 80 and older (24). The consensus view is that cortical microbleeds tend to be manifestations of cerebral amyloid angiopathy, while deep hemisphere lesions are indicative of hypertensive arteriopathy (24). The underlying pathological substrate has been thought to depend on small arterial tears (25), although capillary-derived hemorrhage has been suggested as an alternate source (26).

There is a powerful relationship between cerebral microbleeds and ICH. Prevalence of microbleeds in subjects with spontaneous ICH is 68% (27), and up to 64% among ICH subjects and coexisting TIA/ischemic stroke (28). It is unclear whether microbleeds are the source of these hemorrhages or simply reflect another form of vascular injury predisposing to clinical hemorrhage.

Given these high prevalence rates, the relationship between microbleeds and ICH among patients using anticoagulants becomes of great interest. The importance of this issue was emphasized by the report of Lee et al (29), in which presence of microbleeds appeared to confer a more than 83-fold increased risk of warfarin-associated ICH; number of microbleeds was also associated with hemorrhage risk, as was location (lobar, basal ganglia, and cerebellum). This study was based on a cross-sectional case-control investigation, in which microbleeds associated with intracerebral hemorrhage were excluded. This report was notable as a stunningly high risk assessment for microbleeds in this population, raising the possibility that presence of microbleeds could be an important marker for warfarin-associated ICH. Note, however, the large confidence intervals (5.96–1,159.10) for the findings of the yet-to-be reproduced 83-fold increased risk. Another report, by Soo et al. (30), explored the relationship between numbers of microbleeds and ICH risk, and found that those patients with five or more microbleeds were at highest risk for ICH (7.6%), compared to those with zero, one, and two to four microbleeds (0.6, 1.9, and 4.6%, respectively); however, the overwhelming majority of these patients were using platelet medications rather than anticoagulants.

The microbleeds-ICH risk has been studied in a series of systematic reviews. Presence of microbleeds in warfarin-associated ICH was significantly increased (odds ratio 2.7), compared to ICH without antithrombotic usage (platelet agents or anticoagulants) (28). Initial pooled prospective studies reported microbleeds conferring more than 12-fold increased risk of ICH with use of antithrombotics (28).

More recent estimates of microbleed risk, based on pooled prospective studies, report enhanced risk for ICH (odds ratio 8.5) in TIA-ischemic stroke patients with microbleeds (compared to those without microbleeds) (31). The risk for ICH (enhanced more than 10-fold) was concentrated among Asian patients (31). Prospective risk of ICH specifically among warfarin users with microbleeds was based on pooled data including only five subjects with ICH (odds ratio 3.0, *p* = 0.23) (27).

In summary, microbleeds often coexist with ICH and clearly have predictive value for future ICH. Use of antithrombotic therapies confers additional risk of subsequent ICH on patients with microbleeds. Race appears to have a substantial impact on this risk, and the relative impact of anticoagulants vs. platelet agents on this hemorrhage risk is unclear, as is the role of newer anticoagulants.

## White Matter Disease of Aging and Risk of Intracerebral Hemorrhage

Age-related white matter changes are demonstrable by MRI in more than 95% of the population age 65 or older (32). The more severe form of these changes, sometimes termed leukoaraiosis, are strongly related to presence of cerebral microbleeds (33–37), suggesting a common microvascular etiology for both (38). Presence of leukoaraiosis, as demonstrated by CT, has been shown to substantially predict warfarin-related ICH with odds ratios ranging from 2.7 to 12.9 (39, 40). Predictive value of white matter disease shown by MRI, for anticoagulant-related ICH, is not known.

## MRI Screening for Microbleeds in Anticoagulation Patients

Coexistence of ischemic and hemorrhagic cerebrovascular disorders creates new challenges in clinical neurology, creating the need for a diagnostic paradigm such as “mixed cerebrovascular disease” (41) and requiring novel strategies. The clinical problems inherent in anticoagulation for atrial fibrillation substantially reflect the difficulties raised by mixed cerebrovascular disease. Presence of cerebral microbleeds in this population, combined with the need for anticoagulation to prevent ischemic stroke, creates a combination of ischemic and hemorrhagic cerebrovascular processes that can be unsettling for the clinician.

As noted previously, fear of ICH is a principal driving force in anticoagulation decision-making. MRI screening of these patients therefore may have some appeal as a means for addressing concerns of clinicians faced with the risk of cardioembolic stroke and concurrent risk of brain hemorrhage. If MRI were to be used for these patients, how would the information so obtained be incorporated into clinical practice?

**Figure 1:**
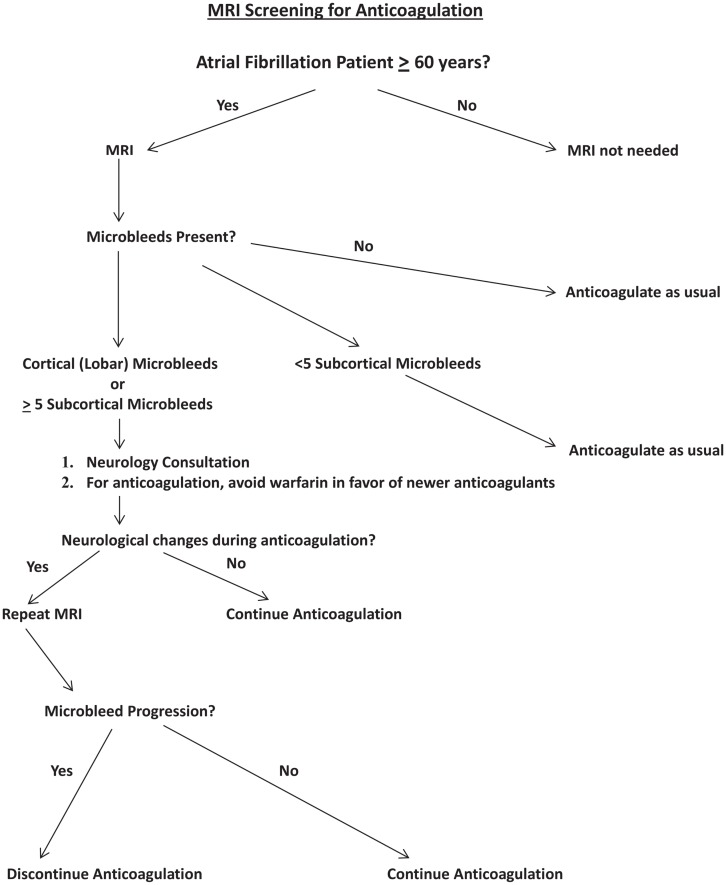
**Proposed algorithm for incorporation of MRI screening into decision-making for anticoagulation for atrial fibrillation patients**. “Newer anticoagulants” refers to agents such as dabigatran and rivaroxaban.

A clinical algorithm that incorporates MRI screening into anticoagulation decision-making for atrial fibrillation patients is proposed (Figure 1). In this pathway, MRI screening is reserved for patients 60 years and older, an age range in which microbleeds prevalence overall is expected to be approximately 20% or more (24). Among those patients who have microbleeds demonstrable on MRI, a distinction is made between cortical microbleeds vs. subcortical microbleeds, and between findings of five or more subcortical microbleeds vs. less than five subcortical microbleeds. The rationale for these distinctions is based on the likelihood of cerebral amyloid angiopathy with presence of cortical microbleeds (24), and the findings of increasing risk of intracerebral hemorrhage with increased numbers of microbleeds (29, 30). Nevertheless, the data on which this algorithm is based are fragmentary, necessitating a considerable amount of speculative extrapolation for constructing the algorithm.

From the perspective of this algorithm, presence of cortical microbleed(s) or at least five subcortical microbleeds would trigger formal neurological consultation, to carefully focus on mental status, gait status, assessment of what kind of supervision may be necessary if the patient began anticoagulants, and to establish a neurological baseline for future comparisons. Anticoagulation with warfarin would be avoided, but new anticoagulants would be acceptable. Any neurological changes while anticoagulated would trigger follow-up MRI to help determine cause of progression and address potential microbleed changes, including size and number of microbleeds (42).

Nevertheless, such an algorithm is likely to be criticized as lacking a substantial foundation of compelling data. Specifically, the relationships between site and numbers of microbleeds (on the one hand) and future bleeding risk (on the other hand) are not that well worked out, and it has not yet been demonstrated that the newer anticoagulants specifically have reduced risk of ICH in patients with cerebral amyloid angiopathy. Indeed, one might simply argue that given the overwhelming benefit of anticoagulation in atrial fibrillation that has already been demonstrated even in an elderly population (43), why is such an algorithm needed? Why not simply move away from warfarin to newer anticoagulants and initiate anticoagulation based on ischemic stroke risk (44)? While there is a compelling logic to these questions, they do not address the fundamental problem described earlier, i.e., the problem of substantial numbers of clinicians avoiding anticoagulation due to excessive fear of ICH. Risk in individual patients is not directly addressed by the reported overall reduced risk of ICH with newer anticoagulants (16, 17). In this context, MRI becomes perhaps the only means by which those concerns may be directly addressed in a systematic manner.

## MRI Screening: Costs and Benefits

Any incorporation of MRI into routine clinical evaluation of atrial fibrillation patients will raise issues of the economics involved. A formal cost-benefit analysis for MRI screening is beyond the scope of this paper. However, some initial observations can be provided. Average cost of brain MRI has been reported as $2700 (45), but actual reimbursement by Medicare or other third parties is considerably lower; for example, Medicare reimbursement for a non-contrast MRI is estimated at just under $500 per scan (www.palmettogba.com).

The population of atrial fibrillation patients in the US has been estimated at 2.3 million (46). It is has been calculated that expanding anticoagulation usage, to one-half of currently non-anticoagulated patients, would result in annual savings of $1.3 billion (46). Given an at-risk population of atrial fibrillation patients aged 60 or older conservatively estimated at two million, justification for MRI screening of this population might require an unrealistic expansion of anticoagulation usage.

However, Mercaldi et al. report estimated savings associated with use of warfarin in patients with atrial fibrillation at more than $9800 per patient per year (47). With costs of MRI scans at Medicare rates, the analysis becomes more favorable. For example, the cost of the scanning program may be covered if anticoagulant usage was incrementally increased by approximately 5% of patients scanned. Costs of additional neurology consults will also impact this analysis.

The logistical exercise of performing the scans would not be inconsequential, with a conservatively estimated 6,000 MRI scanners in the US (48) needing to study two million patients; this patient population could perhaps be covered with an additional patient studied daily for 1 year. If a substantial proportion of MRI scanners were incapable of performing the appropriate sequences, i.e., gradient echo and/or susceptibility-weighted imaging (49), there would be a commensurate enhancement of this increased workload for those scanners that did have this capability.

This analysis is not intended to be definitive. However, it does suggest that large-scale MRI scanning is not entirely unrealistic from an economic perspective. The logistical issues are substantial, but not necessarily unachievable.

## Conclusion

MRI screening for atrial fibrillation patients, to address prospective risk of ICH, has significant appeal. In the minds of many clinicians, concern for hemorrhagic risk with anticoagulants outweighs benefits of stroke prevention. Systemic bleeding is addressed in current hemorrhage risk stratification systems, and incorporation of MRI findings into formal risk stratification efforts would be welcome.

However, there are a number of factors that suggest a cautious approach to this issue. Potential problems, while not necessarily insurmountable, are also not insignificant. These issues include the logistical demands of large-scale MRI screening of atrial fibrillation patients, the uncertain extent of enhanced usage of anticoagulation resulting from MRI screening, the limited data on risk impact of number and sites of cerebral microbleeds, and the profound costs involved. Ongoing prospective trials are expected to provide definitive guidance (50).

Perhaps the most attractive strategy is a limited incorporation of MRI screening in order to optimize patient selection for chronic anticoagulation. Such an intermediate path would incorporate MRI screening not routinely, but in the settings of patients with intermediate CHADS2 scores (e.g., scores of 1–2), for patients whose fall risks and/or mental status changes creates heightened concerns regarding risk for ICH, and for those clinicians whose concerns for ICH risk typically predominate in their decision-making for anticoagulation recommendations in patients with atrial fibrillation. For these subsets, an algorithm as described herein may be useful for providing a means for systematic evaluation of microbleeds and ICH risk in a patient population that remains undertreated. With this approach, increased utilization of anticoagulation is likely to be incremental but nevertheless beneficial given the devastating consequences of stroke due to atrial fibrillation.

## Conflict of Interest Statement

The author has been a member of speakers’ bureau and has received research support from Boehringer Ingelheim.
